# Complete genome sequence of *Methanothermus fervidus* type strain (V24S^T^)

**DOI:** 10.4056/sigs.1283367

**Published:** 2010-11-20

**Authors:** Iain Anderson, Olivier Duplex Ngatchou Djao, Monica Misra, Olga Chertkov, Matt Nolan, Susan Lucas, Alla Lapidus, Tijana Glavina Del Rio, Hope Tice, Jan-Fang Cheng, Roxanne Tapia, Cliff Han, Lynne Goodwin, Sam Pitluck, Konstantinos Liolios, Natalia Ivanova, Konstantinos Mavromatis, Natalia Mikhailova, Amrita Pati, Evelyne Brambilla, Amy Chen, Krishna Palaniappan, Miriam Land, Loren Hauser, Yun-Juan Chang, Cynthia D. Jeffries, Johannes Sikorski, Stefan Spring, Manfred Rohde, Konrad Eichinger, Harald Huber, Reinhard Wirth, Markus Göker, John C. Detter, Tanja Woyke, James Bristow, Jonathan A. Eisen, Victor Markowitz, Philip Hugenholtz, Hans-Peter Klenk, Nikos C. Kyrpides

**Affiliations:** 1 DOE Joint Genome Institute, Walnut Creek, California, USA; 2 HZI – Helmholtz Centre for Infection Research, Braunschweig, Germany; 3 Los Alamos National Laboratory, Bioscience Division, Los Alamos, New Mexico, USA; 4 DSMZ - German Collection of Microorganisms and Cell Cultures GmbH, Braunschweig, Germany; 5 Biological Data Management and Technology Center, Lawrence Berkeley National Laboratory, Berkeley, California, USA; 6 Oak Ridge National Laboratory, Oak Ridge, Tennessee, USA; 7 University of Regensburg, Archaeenzentrum, Regensburg, Germany; 8 University of California Davis Genome Center, Davis, California, USA

**Keywords:** hyperthermophile, strictly anaerobic, motile, Gram-positive, chemolithoautotroph, *Methanothermaceae*, *Euryarchaeota*, GEBA

## Abstract

*Methanothermus fervidus* Stetter 1982 is the type strain of the genus *Methanothermus*. This hyperthermophilic genus is of a thought to be endemic in Icelandic hot springs. *M. fervidus* was not only the first characterized organism with a maximal growth temperature (97°C) close to the boiling point of water, but also the first archaeon in which a detailed functional analysis of its histone protein was reported and the first one in which the function of 2,3-cyclodiphosphoglycerate in thermoadaptation was characterized. Strain V24S^T^ is of interest because of its very low substrate ranges, it grows only on H_2_ + CO_2_. This is the first completed genome sequence of the family *Methanothermaceae*. Here we describe the features of this organism, together with the complete genome sequence and annotation. The 1,243,342 bp long genome with its 1,311 protein-coding and 50 RNA genes is a part of the *** G****enomic* *** E****ncyclopedia of* *** B****acteria and* *** A****rchaea * project.

## Introduction

Strain V24S^T^ (= DSM 2088 = ATCC 43054 = JCM 10308) is the type strain of *Methanothermus fervidus* [1]. Together with *M. sociabilis*, there are currently two species placed in the genus *Methanothermus*. The strain V24S^T^ was isolated from an anaerobic Icelandic spring [1,2] and *M. sociabilis* from a continental solfatara field in Iceland [2]. Since any attempt to isolate *Methanothermus* from similar places (Italy, the Azores, Yellowstone National Park) was without success, Lauerer *et al*. (1986) have speculated that strains of *Methanothermus* may exist endemically within Iceland [2]. The genus name derives from the Latin word “*methanum*”, methane, and from the Greek adjective “*therme*”, meaning heat, which refers to a methane producing organism living in a hot niche [1]. The species epithet *fervidus* comes from the Latin adjective “*fervidus*”, glowing hot, burning, fervent, because of its growth in almost-boiling water [1]. No further cultivated strains belonging to the species *M. fervidus* have been described so far. Here we present a summary classification and a set of features for *M. fervidus* strain V24S^T^, together with the description of the complete genomic sequencing and annotation.

## Classification and features

**Figure 1:**
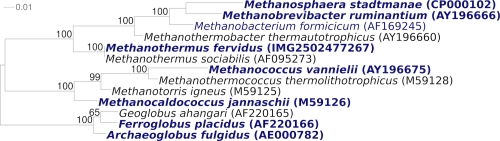
Phylogenetic tree highlighting the position of *M. fervidus* V24S^T^ relative to the other type strains within the family *Methanothermaceae*. The tree was inferred from 1,249 aligned characters [3,4] of the 16S rRNA gene sequence under the maximum likelihood criterion [5] and rooted in accordance with the current taxonomy [6]. The branches are scaled in terms of the expected number of substitutions per site. Numbers above branches are *support values from 100 bootstrap replicates [7] if larger than 60%. Lineages with type strain genome sequencing projects registered in GOLD [8] are shown in blue, published genomes in bold [9-12].

The original 16S rRNA gene sequence of strain V24S^T^ (M59145) shows 92% sequence identity with the 16S rRNA gene of *M. sociabilis* (AF095273) [2] (Figure 1) and 88% identity with an uncultured clone, NRA12 (HM041913). The highest sequence similarities of the strain V24S^T^ 16S rRNA to metagenomic libraries (env_nt) were 87% or less (status August 2010), indicating that members of the species, genus and even family are poorly represented in the habitats screened so far. The 16S rRNA gene sequence of strain V24S^T^ was compared with the most recent release of the Greengenes database using BLAST [13] and the relative frequencies weighted by BLAST scores, of taxa and keywords within the 250 best hits were determined. The five most frequent genera were *Methanobacterium* (55.3%), *Methanothermobacter* (23.5%), *Methanobrevibacter* (12.8%), *Methanothermus* (5.7%) and *Thermococcus* (1.7%). The five most frequent keywords within the labels of environmental samples which yielded hits were 'anaerobic' (7.1%), 'sludge' (4.7%), 'microbial' (3.7%), 'archaeal' (3.5%) and 'temperature' (3.4%). Besides 'sludge', these keywords fit well to what is known from the taxonomy, ecology, and physiology of strain V24S^T^. Environmental samples which yielded hits of a higher score than the highest scoring species were not found. The genome of *M. fervidus* contains two rRNA operons. One of these operons has a closely linked 7S RNA gene, encoding the RNA component of signal recognition particle [14].

Figure 1 shows the phylogenetic neighborhood of *M. fervidus* V24S^T^ in a 16S rRNA based tree. The sequences of the two 16S rRNA gene copies in the genome of *Methanothermus fervidus* DSM 2088 differ from each other by up to four nucleotides, and differ by up to 17 nucleotides from the previously published 16S rRNA sequence (M59145), which contains 87 ambiguous base calls.

**Figure 2:**
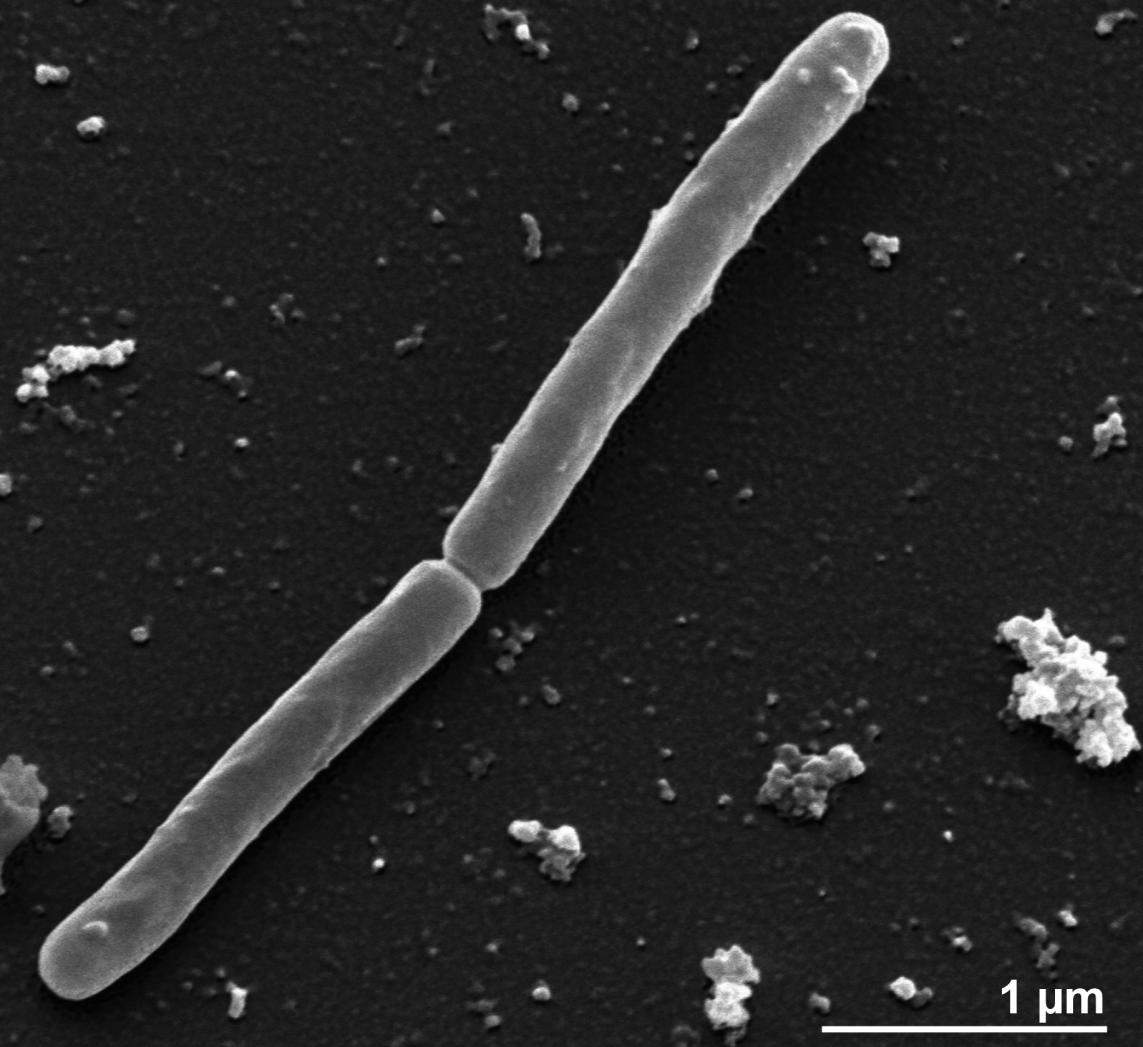
Scanning electron micrograph of *M. fervidus* V24S^T^

Although the cells of the strain V24ST do not contain a typical bacterial peptidoglycan, they stain Gram-positive. Cells are curved rods, 1-3 µm long and 0.3 - 0.4 µm in width (Figure 2 and Table 1), occurring singly and in pairs, with a doubling time of 170 minutes [1]. Round, smooth, opaque, and slightly grayish colonies of 1 to 3 mm in diameter were observed on modified MM-medium plates containing trace amounts of solid sodium dithionite, sodium silicate solution and resazurin   [1]. Strain V24S^T^ is strictly anaerobic and strictly autotrophic [26]. Due to the low melting point of agar, strain V24S^T^ could do not be grown on agar. Cells did not grow at temperatures below 61°C or above 97°C; the optimal temperature was 83°C [1]. Growth occurs at a slightly acidic pH and equal to 6.5, while no growth could be observed at pH above 7.0 [1]. In comparison, *M. sociabilis* grows at the temperatures ranged between 65°C and 97°C, with the optimal temperature at 88°C, its pH for growth being acidic to neutral (pH 5.5 to 7.5) [27]. Strain V24S^T^ produces methane from H_2_ + CO_2_, whereas acetate and formate are not used [1,27]. The addition of 2-mercaptoethanesulfonic acid (coenzyme M) enhances growth, especially when small inoculates are used [1]. In artificial medium, yeast extract is required as an organic factor for growth [1]. Strain V24S^T^ gains energy by oxidizing H_2_ to reduce CO_2_ as the terminal electron acceptor [26,28]. At the time of isolation, strain V24S^T^ was described to be nonmotile [1]. Later, strain V24S^T^ as well as *M. sociabilis* were described to be motile *via* bipolar peritrichous ‘flagella’, which was taken to indicate motility [28]. These cell surface appendages, however, were recently determined to have a diameter of 5-6 nm, and therefore, very probably, represent not organelles used for motility, but for adhesion [R Wirth *et al.,* unpublished]. The genome does not contain any flagellar genes. *M. fervidus* produces large intracellular potassium concentrations and amounts of 2,3-cyclic diphosphoglycerate, which are both thought to be involved in the thermoadaptation of *M. fervidus* [29,30]. Moreover, the DNA-binding protein HMf (histone *M. fervidus*), which binds to double stranded DNA molecules and increases their resistance to thermal denaturation, has been of interest in *M. fervidus* [31]. A partial amino acid sequence analysis of the D-glyceraldehyde-3-phosphate dehydrogenase of *M. fervidus* shows high sequence similarity to the enzymes from eubacteria and from the cytoplasm of eukaryotes [32]. This enzyme reacts with both NAD^+^ and NADP^+^ [32,33] and is not inhibited by pentalenolactone [32]. However, the enzyme activity is low at temperatures below 40°C, but it is intrinsically stable only up to 75°C [32], which is interesting as growth of *M. fervidus* may occur up to 97°C [1]. Also, the biochemistry of triose-phosphate isomerase, which catalyzes the interconversion of dihydroxyacetone phosphate (DHAP) and glyceraldehyde 3-phosphate (GAP) in the reversible Embden-Meyerhof-Parnas (EMP) pathway, has been studied to some detail in *M. fervidus* [34].

**Table 1:** Classification and general features of *M. fervidus* V24S^T^ according to the  MIGS recommendations [15]

**MIGS ID**	**Property**	**Term**	**Evidence code**
	Current classification	Domain *Archaea*	TAS [16]
		Phylum *Euryarchaeota*	TAS [17,18]
		Class *Methanobacteria*	TAS [18,19]
		Order *Methanobacteriales*	TAS [20-22]
		Family *Methanothermaceae*	TAS [1,23]
		Genus *Methanothermus*	TAS [1,23]
		Species *Methanothermus fervidus*	TAS [1,23]
		Type strain V24S	TAS [1]
	Gram stain	positive	TAS [1]
	Cell shape	straight to curved, single and in pair rods	TAS [1]
	Motility	non-motile	TAS [1]
	Sporulation	not reported	NAS
	Temperature range	61°C–97°C	TAS [1]
	Optimum temperature	83°C	TAS [1]
	Salinity	not reported	NAS
MIGS-22	Oxygen requirement	strict anaerobic	TAS [1]
	Carbon source	CO_2_	TAS [1]
	Energy source	H_2_ + CO_2_	TAS [1]
MIGS-6	Habitat	solfataric fields	TAS [1]
MIGS-15	Biotic relationship	not reported	NAS
MIGS-14	Pathogenicity	no	NAS
	Biosafety level	1	TAS [24]
	Isolation	Icelandic hot spring	TAS [1]
MIGS-4	Geographic location	Kerlingarfjöll mountains, Iceland	TAS [1]
MIGS-5	Sample collection time	1979	NAS
MIGS-4.1 MIGS-4.2	Latitude Longitude	64.65 19.25	NAS
MIGS-4.3	Depth	surface	TAS [1]
MIGS-4.4	Altitude	1.477 m	NAS

Evidence codes - IDA: Inferred from Direct Assay (first time in publication); TAS: Traceable Author Statement (i.e., a direct report exists in the literature); NAS: Non-traceable Author Statement (i.e., not directly observed for the living, isolated sample, but based on a generally accepted property for the species, or anecdotal evidence). These evidence codes are from of the Gene Ontology project [25]. If the evidence code is IDA, then the property was directly observed by one of the authors or an expert mentioned in the acknowledgements

### Chemotaxonomy

The cell envelope of the strain V24S^T^ consists of a double-layer of pseudomurein and protein, while the cell wall contains pseudomurein consisting of N-acetyl-glucosamine, N-acetyl-galactosamine, N-talosaminuronic acid, glutamic acid, alanine, and lysine [1,2].

*M. fervidus* contains approximately 50% diethers, 25% diglycerol tetraethers and 25% of an unknown component moving slower than the tetraethers when examined by thin layer chromatography [2]. Here, *M. fervidus* differs from *M. sociabilis*, which lacks the unknown component while its diether and tetraethers were found at about equal proportions [2]. The diethers of *M. fervidus* contain only C_20_ phytanyl chains while the tetraethers include about 98-99% C_40_ biphytane and only some trace of C_40_ monocyclic biphytane [2]. Besides an unknown core lipid (FU, 31% of total core lipids, migrates slower than caldarchaeol by thin layer chromatography), other core lipids found in *M. fervidus* were caldarchaeol (60%), archaeol (4%) and others (5%) [35,36]. Interestingly, *M. fervidus* also differs from *M. sociabilis* regarding the glycolipid composition with four glycolipids and about equal proportions of five phospholipids and only three phospholipids for *M. sociabilis* [2].

## Genome sequencing and annotation

### Genome project history

This organism was selected for sequencing on the basis of its phylogenetic position [37], and is part of the *** G****enomic* *** E****ncyclopedia of* *** B****acteria and* *** A****rchaea * project [38]. The genome project is deposited in the Genome OnLine Database [8] and the complete genome sequence is deposited in GenBank. Sequencing, finishing and annotation were performed by the DOE Joint Genome Institute (JGI). A summary of the project information is shown in Table 2.

**Table 2:** Genome sequencing project information

**MIGS ID**	**Property**	**Term**
MIGS-31	Finishing quality	Finished
MIGS-28	Libraries used	Three genomic libraries: one 454 pyrosequence standard library, one 454 PE library (17.8 kb insert size), one Illumina library
MIGS-29	Sequencing platforms	Illumina GAii, 454 GS FLX
MIGS-31.2	Sequencing coverage	530 × Illumina; 75.0 × pyrosequence
MIGS-30	Assemblers	Newbler version 2.0.00.20-PostRelease- 11-05-2008-gcc-3.4.6, phrap
MIGS-32	Gene calling method	Prodigal 1.4, GenePRIMP
	INSDC ID	CP002278
	Genbank Date of Release	November 5, 2010
	GOLD ID	Gc01509
	NCBI project ID	33689
	Database: IMG-GEBA	2502422313
MIGS-13	Source material identifier	DSM 2088
	Project relevance	Tree of Life, GEBA

### Growth conditions and DNA isolation

*M. fervidus* V24S^T^, DSM 2088, was grown anaerobically in culture vessels made of type III glass (alkali-rich soda lime glass) in DSMZ medium 203 (*M. fervidus* medium) [39] at 83°C. DNA was isolated from 0.5-1 g of cell paste using Qiagen Genomic 500 DNA Kit (Qiagen, Hilden, Germany) following the standard protocol as recommended by the manufacturer, with a modified cell lysis step. The modified lysis mixture contained only 100 µl lysozyme, but additional 58 µl achromopeptidase, lysostaphine, mutanolysin, each, for over night incubation at 35°C on a shaker. Proteinase K digestion was reduced to 200 µl for 1h 37°C.

### Genome sequencing and assembly

The genome was sequenced using a combination of Illumina and 454 sequencing platforms. All general aspects of library construction and sequencing can be found at the JGI website [40]. Pyrosequencing reads were assembled using the Newbler assembler version 2.0.00.20-PostRelease-11-05-2008-gcc-3.4.6 (Roche). The initial Newbler assembly consisting of 24 contigs in one scaffold was converted into a phrap assembly [41] by making fake reads from the consensus, collecting the read pairs in the 454 paired end library. Illumina GAii sequencing data (636 Mb) was assembled with Velvet [42] and the consensus sequences were shredded into 1.5 kb overlapped fake reads and assembled together with the 454 data. 454 Draft assembly was based on 96.5.0 Mb 454 draft data and all of the 454 paired end data. Newbler parameters are -consed -a 50 -l 350 -g -m -ml 20. The Phred/Phrap/Consed software package [41] was used for sequence assembly and quality assessment in the subsequent finishing process. After the shotgun stage, reads were assembled with parallel phrap (High Performance Software, LLC). Possible mis-assemblies were corrected with gapResolution [40], Dupfinisher, or sequencing cloned bridging PCR fragments with subcloning or transposon bombing (Epicentre Biotechnologies, Madison, WI) [43]. Gaps between contigs were closed by editing in CONSED and additional sequencing reactions were necessary to close gaps and/or to raise the quality of the finished sequence. Illumina reads were also used to correct potential base errors and increase consensus quality using a software Polisher developed at JGI [44]. The error rate of the completed genome sequence is less than 1 in 100,000. Together, the combination of the Illumina and 454 sequencing platforms provided 605 × coverage of the genome. The final assembly contained 267,328 pyrosequence and 17,666,667 Illumina reads.

### Genome annotation

Genes were identified using Prodigal [45] as part of the Oak Ridge National Laboratory genome annotation pipeline, followed by a round of manual curation using the JGI GenePRIMP pipeline [46]. The predicted CDSs were translated and used to search the National Center for Biotechnology Information (NCBI) nonredundant database, UniProt, TIGRFam, Pfam, PRIAM, KEGG, COG, and InterPro databases. Additional gene prediction analysis and functional annotation was performed within the Integrated Microbial Genomes - Expert Review (IMG-ER) platform [47].

## Genome properties

**Figure 3:**
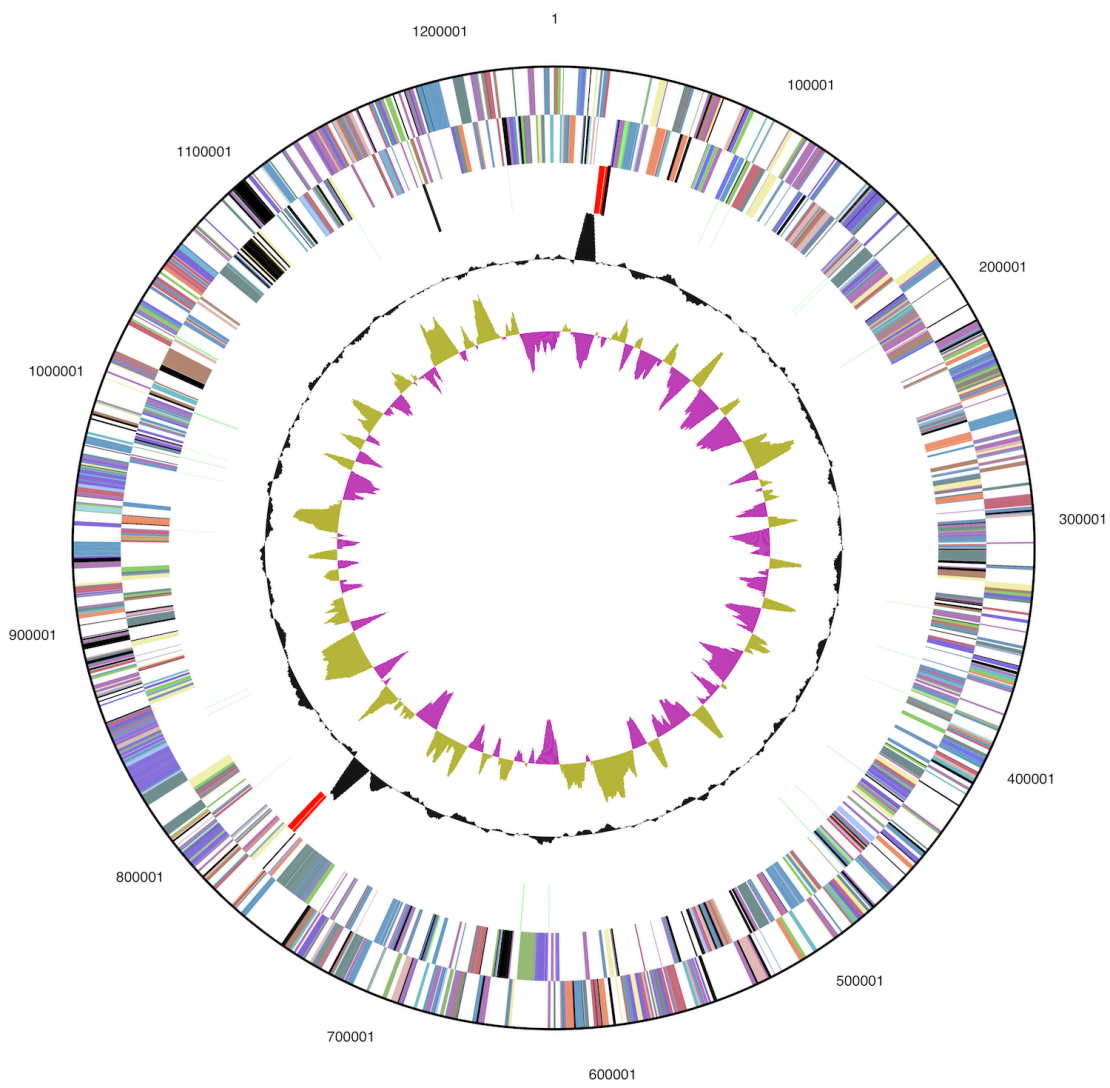
Graphical circular map of the genome. From outside to the center: Genes on forward strand (color by COG categories), Genes on reverse strand (color by COG categories), RNA genes (tRNAs green, rRNAs red, other RNAs black), GC content, GC skew.

The genome consists of a 1,243,342 bp long chromosome with a 31.6% GC content (Table 3 and Figure 3). Of the 1,361 genes predicted, 1,311 were protein-coding genes, and 50 RNAs; twenty eight pseudogenes were also identified. The majority of the protein-coding genes (74.8%) were assigned with a putative function while the remaining ones were annotated as hypothetical proteins. The distribution of genes into COGs functional categories is presented in Table 4.

**Table 3:** Genome Statistics

**Attribute**	**Value**	**% of Total**
Genome size (bp)	1,243,342	100.00%
DNA coding region (bp)	1,163,294	93.56%
DNA G+C content (bp)	393,356	31.64%
Number of replicons	1	100.00%
Extrachromosomal elements	0	
Total genes	1,361	100.00%
RNA genes	50	3.67%
rRNA operons	2	0.15%
Protein-coding genes	1,311	96.33%
Pseudo genes	28	2.06%
Genes with function prediction	1,018	74.80%
Genes in paralog clusters	100	7.35%
Genes assigned to COGs	1,126	82.73%
Genes assigned Pfam domains	1,144	84.06%
Genes with signal peptides	109	8.01%
Genes with transmembrane helices	248	18.22%
CRISPR repeats	0	

**Table 4:** Number of genes associated with the general COG functional categories

**Code**	**value**	**% age**	**Description**
J	144	12.2	Translation, ribosomal structure and biogenesis
A	2	0.2	RNA processing and modification
K	47	4.0	Transcription
L	49	4.2	Replication, recombination and repair
B	4	0.3	Chromatin structure and dynamics
D	12	1.0	Cell cycle control, cell division, chromosome partitioning
Y	0	0.0	Nuclear structure
V	6	0.5	Defense mechanisms
T	11	0.9	Signal transduction mechanisms
M	52	4.4	Cell wall/membrane/envelope biogenesis
N	1	0.1	Cell motility
Z	0	0.0	Cytoskeleton
W	0	0.0	Extracellular structures
U	14	1.2	Intracellular trafficking and secretion, and vesicular transport
O	46	3.9	Posttranslational modification, protein turnover, chaperones
C	111	9.4	Energy production and conversion
G	39	3.3	Carbohydrate transport and metabolism
E	88	7.5	Amino acid transport and metabolism
F	47	4.0	Nucleotide transport and metabolism
H	115	9.8	Coenzyme transport and metabolism
I	17	1.4	Lipid transport and metabolism
P	50	4.2	Inorganic ion transport and metabolism
Q	5	0.4	Secondary metabolites biosynthesis, transport and catabolism
R	172	14.6	General function prediction only
S	147	12.5	Function unknown
-	235	17.3	Not in COGs
